# GLP-1 and dual GIP/GLP-1 agonists in obese patients with HFpEF: a systematic review and meta-analysis of RCTs

**DOI:** 10.1186/s12872-026-05808-7

**Published:** 2026-03-30

**Authors:** Elhaitham Yasir Awad Musa, Amar Yasir Awad Musa

**Affiliations:** National Ribat University, Khartoum, Sudan

**Keywords:** Heart failure with preserved ejection fraction, Obesity, GLP-1 receptor agonist, Tirzepatide, Semaglutide, Meta-analysis, Systematic review, HFpEF

## Abstract

**Background:**

Obesity-related heart failure with preserved ejection fraction (HFpEF) is characterized by impaired exercise tolerance and poor health status. Metabolic modulation using glucagon-like peptide-1 (GLP-1) receptor agonists and dual glucose-dependent insulinotropic polypeptide (GIP)/GLP-1 agonists has emerged as a potential therapeutic strategy.

**Objectives:**

To evaluate effects of GLP-1 and GIP/GLP-1 agonists on heart failure hospitalization, symptoms, physical function, body weight, and mortality in patients with HFpEF and obesity.

**Methods:**

We searched MEDLINE, CENTRAL, and ClinicalTrials.gov through November 2025 for randomized controlled trials enrolling adults with HFpEF (left ventricular ejection fraction ≥ 45%) and obesity (body mass index ≥ 30 kg/m², or ≥ 27 kg/m² with at least one obesity-related comorbidity) treated with GLP-1 or GLP-1/GIP agonists. Primary outcome was first heart failure hospitalization. Secondary outcomes included Kansas City Cardiomyopathy Questionnaire Clinical Summary Score, six-minute walk distance, percentage bodyweight change, and all-cause mortality.

**Results:**

Four trials (*n* = 4,149) met criteria. Two trials contributed adjudicated first heart failure hospitalization, demonstrating pooled hazard ratio 0.52 (95% confidence interval 0.33–0.82). Incretin therapy improved Kansas City Cardiomyopathy Questionnaire by 7.4 (95% CI 4.9–9.9) points and six-minute walk distance by 17.6 m (95% CI 10.7–24.5). Body weight decreased by -9.6% (95% CI -11.3 to -8.0). All-cause mortality did not differ significantly (hazard ratio 0.90, 95% confidence interval 0.67–1.22); however, given the limited number of events across available trials, this finding should be interpreted as inconclusive rather than indicative of a null mortality effect.

**Conclusions:**

GLP-1-based and dual GIP/GLP-1 therapies improve symptoms, physical function, and weight and reduce heart failure events in adults with obesity-associated HFpEF, supporting metabolic modulation as a therapeutic approach.

**Trial registration:**

Systematic review registration: CRD420251237462.

**Supplementary Information:**

The online version contains supplementary material available at 10.1186/s12872-026-05808-7.

## Introduction

Heart failure with preserved ejection fraction (HFpEF) comprises approximately half of all heart failure cases and is closely linked to cardiometabolic dysfunction, systemic inflammation, and obesity [1]. Individuals with obesity-related HFpEF often exhibit marked impairments in exercise tolerance, elevated filling pressures, and diminished health status, reflecting the combined hemodynamic and metabolic burden of excess adiposity [1]. Despite its growing prevalence, HFpEF has historically lacked robust evidence-based therapies with proven impact on clinical outcomes. Obesity contributes mechanistically to HFpEF through increased visceral adiposity, myocardial and pericardial fat deposition, arterial and ventricular stiffness, impaired nitric oxide bioavailability, and abnormalities in ventricular-vascular coupling [1]. Among HFpEF phenotypes, obesity-related HFpEF is increasingly recognized as a distinct cardiometabolic subtype with high symptom burden and substantial therapeutic need [1]. Glucagon-like peptide-1 receptor agonists and dual glucose-dependent insulinotropic polypeptide/GLP-1 agonists produce clinically meaningful weight loss, improve glycemic control, and reduce inflammation and cardiometabolic risk [2]. Recently, randomized controlled trials specifically evaluating these agents in HFpEF—SUMMIT (tirzepatide), STEP-HFpEF, and STEP-HFpEF DM (semaglutide)—have demonstrated improvements in health status, physical function, and cardiometabolic markers. The SELECT cardiovascular outcomes trial included a prespecified HFpEF subgroup with adjudicated heart failure outcomes, providing complementary data in this population.

A recent systematic review by Waqas et al. [3] evaluated GLP-1 receptor agonists in HFpEF but did not restrict to obesity-related HFpEF, did not prespecify first adjudicated heart failure hospitalization as the primary outcome, and did not integrate functional capacity or weight-change data across trials. No prior systematic review has restricted inclusion to trials requiring obesity while prespecifying first heart failure hospitalization as the primary outcome. This approach addresses two methodological gaps. First, obesity-related HFpEF represents a mechanistically distinct phenotype characterized by metabolic dysfunction and inflammation that may respond preferentially to incretin-based therapies targeting these pathways [1]. Including trials without obesity requirements risks diluting treatment effects specific to this population. Second, first heart failure hospitalization is a patient-centered endpoint that captures the initial decompensation event and directly informs clinical decision-making, whereas composite endpoints including recurrent events introduce analytical complexity. By focusing on trials enrolling patients with obesity (BMI ≥ 30 kg/m²) and examining first hospitalization, we provide phenotype-specific evidence relevant to contemporary clinical practice.

This systematic review examines the effects of GLP-1-based and dual GIP/GLP-1 incretin therapies on first heart failure hospitalization, health status (KCCQ), functional capacity, body weight, and mortality in adults with HFpEF and obesity. Our goal is to integrate data across trials and provide clarity on their clinical utility and potential role in HFpEF management.

## Methods

### Search strategy

We conducted a systematic search of MEDLINE (via PubMed), the Cochrane Central Register of Controlled Trials (CENTRAL), Epistemonikos, ClinicalTrials.gov, and the WHO International Clinical Trials Registry Platform (ICTRP) from inception through November 2025. We additionally hand-searched five major cardiology journals indexed by Scopus due to access limitations (JACC, JAMA Cardiology, Circulation, European Heart Journal, and Nature Reviews Cardiology). No language restrictions were applied. The full search strategy (Supplementary Appendix 2) uses GLP-1 receptor agonist MeSH terms alongside semaglutide, tirzepatide, and GLP-1 as text terms, combined with HFpEF and heart failure with preserved ejection fraction terminology. The search adheres to PRISMA 2020 recommendations [4]. A PRISMA flow diagram summarizing the identification, screening, eligibility assessment, and inclusion of studies is provided in Supplementary Appendix 1.

### Eligibility criteria

Eligible studies met all of the following prespecified criteria:

1. randomized controlled trial.
2. adult participants with heart failure with preserved ejection fraction (HFpEF), defined as left ventricular ejection fraction LVEF ≥ 45%. We selected an LVEF threshold of ≥ 45% to align with contemporary HFpEF definitions and to avoid excluding earlier trials that prespecified analyses using this cut-off.
3. participants with obesity (BMI ≥ 30 kg/m² or ≥ 27 kg/m² with at least one obesity-related comorbidity).
4. intervention using any glucagon-like peptide-1 receptor agonist (GLP-1 RA) or dual glucose-dependent insulinotropic polypeptide (GIP)/GLP-1 receptor agonist. Trials were eligible irrespective of the specific agent within the class; semaglutide and tirzepatide were the only agents with HFpEF-specific randomized trial data identified.
5. availability of at least one of the following outcomes: HF hospitalization, KCCQ-CSS, 6MWD, percentage weight change, all-cause mortality, or composite cardiovascular death or HHF.

Trials enrolling mixed HF phenotypes were eligible only if HFpEF subgroup data were fully extractable. One full-text report was excluded because the overall trial population did not meet HFpEF criteria. However, a prespecified HFpEF subgroup from the same study provided eligible data and was included.

Trials not reporting adjudicated HF endpoints separately (e.g., STEP-HFpEF for HF hospitalization) were included for symptom, weight, and functional outcomes but excluded from the HF hospitalization meta-analysis.

### Outcomes

The primary outcome for this review was first heart failure hospitalization. Secondary outcomes included: (1) change in KCCQ-CSS; (2) change in 6-minute walk distance (6MWD); (3) percentage change in bodyweight; (4) all-cause mortality; and (5) composite cardiovascular death or HF events.

### Data extraction

Two reviewers independently extracted data from each included study, including baseline characteristics, intervention details, and all prespecified outcomes. HF hospitalization and composite HF event definitions were taken exactly as reported in each trial’s protocol-specified endpoint hierarchy. Disagreements were resolved by consensus.

### Risk of bias assessment

Risk of bias was assessed independently by two reviewers using the Cochrane Risk of Bias tool version 2 (RoB 2) [5]. Domains included: randomization process, deviations from intended interventions, missing outcome data, outcome measurement, and selective reporting. Detailed domain-level judgments and justifications are available in Supplementary Appendix 3/3B.

### Certainty of evidence (GRADE)

Certainty of evidence for each outcome was rated using GRADE methodology [6]. The full GRADE Summary of Findings table (Appendix 5 A) and evidence profile (Appendix 5B) include assessments of risk of bias, inconsistency, indirectness, imprecision, and publication bias.

### Statistical analysis

Continuous outcomes were analyzed as mean differences (MDs) using change-from-baseline values when available. All three trials contributing continuous outcomes (SUMMIT, STEP-HFpEF, STEP-HFpEF DM) reported treatment effects as estimated between-group mean differences at week 52 from analysis of covariance models. These were extracted directly as change-from-baseline differences with their reported 95% confidence intervals. No SDs required imputation; where standard errors were reported, conversion used published sample sizes. Time-to-event outcomes were synthesized using hazard ratios (HRs). We initially prespecified fixed-effect models owing to the small number of trials per outcome. However, upon extracting data, we identified substantial between-trial heterogeneity in design characteristics (Table 1), including differences in LVEF thresholds (≥ 45% vs. ≥ 50%), follow-up duration (52 weeks to 39.8 months), and population characteristics (diabetes status, obesity severity, baseline functional impairment). This constitutes a prespecified protocol deviation: fixed-effect models were originally planned but the approach was changed to random-effects meta-analysis after data extraction revealed substantial between-trial heterogeneity in design characteristics (Table 1). This decision was made prior to examining effect sizes, based solely on observed differences in LVEF thresholds, follow-up duration, diabetes prevalence, and obesity severity across included trials. Accordingly, we used random-effects meta-analysis with the DerSimonian-Laird method to estimate between-study variance (τ²). For outcomes with three or more studies, we applied the Hartung-Knapp-Sidik-Jonkman (HKSJ) variance correction to provide more appropriate confidence intervals that account for uncertainty in τ² estimation given the small number of studies. For outcomes with only two studies, we used standard random-effects methods, as the HKSJ adjustment with two studies (one degree of freedom) tends to produce overly conservative confidence intervals [7]. Between-trial heterogeneity was assessed using the I² statistic, with values of 25%, 50%, and 75% representing low, moderate, and high heterogeneity, respectively. We calculated τ² (between-study variance) for all outcomes and Cochran’s Q test to assess statistical heterogeneity. Forest plots for all outcomes are presented in Supplementary Appendix 6. 

Trial-level effect estimates for all prespecified outcomes are summarized in (Table 2).

**Table 1:** Key Design and Population Characteristics of Included Trials

Characteristic	SUMMIT (tirzepatide)	STEP-HFpEF (semaglutide)	STEP-HFpEF DM (semaglutide)	SELECT HFpEF subgroup (semaglutide)
Clinical setting	Obesity-related HFpEF with functional limitation	Obesity-related HFpEF without diabetes	Obesity-related HFpEF with type 2 diabetes	Overweight/obesity with established ASCVD and prevalent HFpEF
Sample size, randomized (n)	731 (364 tirzepatide; 367 placebo)	529 (263 Semaglutide; 266 placebo)	616 (310 Semaglutide; 306 placebo)	2,273 (1,174 semaglutide; 1,099 placebo)
HF definition / LVEF criterion	Symptomatic HF with LVEF ≥ 50%	Symptomatic HF with LVEF ≥ 45%	Symptomatic HF with LVEF ≥ 45%	Clinician-diagnosed HF; HFpEF subgroup defined by LVEF ≥ 50%
BMI inclusion	BMI ≥ 30 kg/m²	BMI ≥ 30 kg/m²	BMI ≥ 30 kg/m² plus type 2 diabetes	BMI ≥ 27 kg/m² (overall SELECT); HFpEF subgroup meets criteria
Key recent HF decompensation requirement	Hospitalization or urgent visit for HF, elevated natriuretic peptides, or structural heart disease	Elevated natriuretic peptides and structural HFpEF features	Elevated natriuretic peptides, structural HFpEF features or recent HF hospitalization	Prevalent HF adjudicated before randomization
Median/mean follow-up	Median 104 weeks	52 weeks	52 weeks	Median 39.8 months (overall SELECT; HFpEF subgroup similar)

Characteristics of four randomized controlled trials included in the systematic review and meta-analysis. All trials enrolled adults with heart failure with preserved ejection fraction (LVEF ≥ 45–50%) and obesity (BMI ≥ 27–30 kg/m²) as mandatory inclusion criteria. The overall SELECT trial required BMI ≥ 27 kg/m². Within the HFpEF subgroup (*n* = 2,273), approximately 76% had BMI ≥ 30 kg/m² (546 of 2,273 patients had BMI < 30 per Deanfield et al. Table 1). Patients with BMI 27-29.9 were eligible under our prespecified ≥ 27 + comorbidity criterion, with established ASCVD as the qualifying comorbidity. See Limitations for discussion. SUMMIT evaluated tirzepatide (dual GIP/GLP-1 agonist) while the remaining trials evaluated semaglutide (GLP-1 receptor agonist). Follow-up duration ranged from 52 weeks (STEP trials) to a median of 104 weeks (SUMMIT) and 39.8 months (SELECT HFpEF subgroup). Age and BMI were similar across trials. The proportion with type 2 diabetes varied from 0% (STEP-HFpEF) to 100% (STEP-HFpEF DM). All trials were double-blind, placebo-controlled, and employed independent adjudication of cardiovascular outcomes

*6MWD* six-minute walk distance, *BMI* body mass index, *DM* diabetes mellitus, *HFpEF* heart failure with preserved ejection fraction, *KCCQ-CSS* Kansas City Cardiomyopathy Questionnaire Clinical Summary Score, *LVEF* left ventricular ejection fraction, *NYHA* New York Heart Association functional class, *SELECT* Semaglutide Effects on Cardiovascular Outcomes in People with Overweight or Obesity, *STEP-HFpEF* Effect of Semaglutide on Function and Symptoms in Subjects with Obesity-related HFpEF, *SUMMIT* Study of Tirzepatide in Participants with HFpEF and Obesity

**Table 2:** Trial-Level Effects on Prespecified Outcomes

Outcome	Trial	Effect Measure	Estimate	Direction
First HF hospitalization	SUMMIT	HR	0.44 (0.22–0.87)	Favours tirzepatide
First HF hospitalization	SELECT HFpEF subgroup	HR	0.59 (0.31–1.06)	Favours semaglutide
KCCQ-CSS change	SUMMIT	MD	6.9 (3.3–10.6)	Favours tirzepatide
KCCQ-CSS change	STEP-HFpEF	MD	7.8 (4.8–10.9)	Favours semaglutide
KCCQ-CSS change	STEP-HFpEF DM	MD	7.3 (4.1–10.4)	Favours semaglutide
6MWD change (m)	SUMMIT	MD	18.3 (9.9–26.7)	Favours tirzepatide
6MWD change (m)	STEP-HFpEF	MD	20.3 (8.6–32.1)	Favours semaglutide
6MWD change (m)	STEP-HFpEF DM	MD	14.3 (3.7–24.9)	Favours semaglutide
Weight (% change)	SUMMIT	MD	-11.6% (-12.9 to -10.4)	Favours tirzepatide
Weight (% change)	STEP-HFpEF	MD	-10.7% (-11.9 to -9.4)	Favours semaglutide
Weight (% change)	STEP-HFpEF DM	MD	-6.4% (-7.6 to -5.2)	Favours semaglutide
All-cause mortality	SUMMIT	HR	1.25 (0.63–2.45)	Inconclusive.
All-cause mortality	SELECT HFpEF subgroup	HR	0.83 (0.59–1.16)	Inconclusive.
Composite CV death or HF	SUMMIT	HR	0.62 (0.41–0.95)	Favours tirzepatide
Composite CV death or HF	SELECT HFpEF subgroup	HR	0.75 (0.52–1.07)	Favours semaglutide

Summary of treatment effects from individual trials on prespecified outcomes. Effect measures are presented as reported in primary trial publications: hazard ratios (HR) for time-to-event outcomes (first heart failure hospitalization, all-cause mortality, composite cardiovascular death or HF events) and mean differences (MD) for continuous outcomes (KCCQ-CSS, six-minute walk distance, body weight). All trials demonstrated consistent direction of benefit favoring incretin therapy across symptom and functional outcomes. Statistical significance (*p* < 0.05) was achieved for most outcomes except all-cause mortality. SUMMIT and SELECT HFpEF subgroup contributed data for adjudicated first-event heart failure hospitalization, while STEP trials reported HF events using a broader endpoint definition incorporating urgent IV visits in addition to hospitalizations, preventing direct pooling

*CI* confidence interval, *HF* heart failure, *HR* hazard ratio, *KCCQ-CSS* Kansas City Cardiomyopathy Questionnaire Clinical Summary Score, *MD* mean difference, *N/A* not applicable or not reported, *SELECT* Semaglutide Effects on Cardiovascular Outcomes in People with Overweight or Obesity, *STEP-HFpEF* Effect of Semaglutide on Function and Symptoms in Subjects with Obesity-related HFpEF, *STEP-HFpEF DM* Semaglutide Treatment Effect in People with Obesity and HFpEF and Diabetes Mellitus, *SUMMIT* Study of Tirzepatide in Participants with HFpEF and Obesity

HF hospitalization meta-analysis was restricted to trials reporting adjudicated first-event HRs (SUMMIT, SELECT HFpEF subgroup). Symptom and functional outcomes included SUMMIT, STEP-HFpEF, and STEP-HFpEF DM, but not SELECT, as KCCQ and 6MWD were not measured in SELECT HFpEF subgroup. Analyses were performed using standard inverse-variance methods.

## Results

### Study selection

Four randomized controlled trials met the inclusion criteria: SUMMIT [8], STEP‑HFpEF [9], STEP‑HFpEF DM [10], and the HFpEF subgroup of SELECT [11]. Together, these trials randomized 4,149 participants (731 in SUMMIT; 529 in STEP‑HFpEF; 616 in STEP‑HFpEF DM; 2,273 in the SELECT HFpEF subgroup). SUMMIT and SELECT HFpEF subgroup contributed time-to-event outcomes including first heart failure hospitalization, all-cause mortality, and composite cardiovascular death or heart failure outcomes, while SUMMIT, STEP-HFpEF, and STEP-HFpEF DM contributed symptom, functional, and weight outcomes.

Full PRISMA information is provided in the Supplementary Appendix.

### Study characteristics

HFpEF definitions differed between trials. SUMMIT required symptomatic HF with LVEF ≥ 50%. STEP‑HFpEF and STEP‑HFpEF DM required symptomatic HF with LVEF ≥ 45%. SELECT enrolled adults with obesity and cardiovascular disease, and its HFpEF subgroup was defined by clinician-diagnosed HF with LVEF ≥ 50%.

Follow‑up duration was 52 weeks in the STEP trials, a median of 104 weeks in SUMMIT, and 39.8 months in the SELECT HFpEF subgroup. Table 1 summarizes key design and baseline characteristics.

### First heart failure hospitalization

Two trials (SUMMIT, SELECT HFpEF subgroup) reported adjudicated first heart failure hospitalization. In SUMMIT, tirzepatide reduced first HF hospitalization with HR 0.44 (95% CI 0.22–0.87), while in SELECT HFpEF subgroup, semaglutide showed a consistent though non-significant reduction with HR 0.59 (95% CI 0.31–1.06).

Pooled analysis using random-effects meta-analysis demonstrated a significant reduction in first heart failure hospitalization (HR 0.52, 95% CI 0.33–0.82). The SELECT HFpEF contribution to this analysis represents a prespecified but power-limited subgroup of a cardiovascular outcomes trial designed for MACE reduction rather than HF hospitalization. Between-study heterogeneity was minimal (I² = 0.0%, τ² = 0.00, Q = 0.48, *p* = 0.49), indicating consistent effects across trials despite differences in study design. The forest plot is shown in Supplementary Figure S1.

### Composite HF events and cardiovascular outcomes (secondary)

Composite HF endpoints differed between trials and were analyzed separately from first HHF. In STEP-HFpEF and STEP-HFpEF DM, semaglutide reduced adjudicated HF events in exploratory time-to-event analyses (HR 0.08, 95% CI 0.00-0.42 in STEP-HFpEF; HR 0.40, 95% CI 0.15–0.92 in STEP-HFpEF DM). However, their HF event endpoint incorporated urgent outpatient visits requiring intravenous therapy in addition to hospitalizations, making it non-comparable to the hospitalization-only first-event HRs reported in SUMMIT and SELECT HFpEF. Furthermore, given the small number of events (1 vs. 12 in STEP-HFpEF; 7 vs. 18 in STEP-HFpEF DM) and the exploratory status of these analyses, formal pooling was not performed. The consistent directional signal across STEP trials nonetheless supports biological plausibility of the HHF reduction observed in the primary analysis. For the composite outcome of cardiovascular death or heart failure events, two trials (SUMMIT, SELECT HFpEF subgroup) contributed data. Pooled analysis demonstrated a significant reduction (HR 0.69, 95% CI 0.53–0.91). Between-study heterogeneity was minimal (I² = 0.0%, τ² = 0.00, Q = 0.47, *p* = 0.49). The forest plot is shown in Supplementary Figure S6.

### Health status (KCCQ-CSS)

Three trials (SUMMIT, STEP-HFpEF, STEP-HFpEF DM) reported change in Kansas City Cardiomyopathy Questionnaire Clinical Summary Score at 52 weeks. In SUMMIT, tirzepatide improved KCCQ-CSS by 6.9 points (95% CI 3.3–10.6) compared with placebo. In STEP-HFpEF, semaglutide improved KCCQ-CSS by 7.8 points (95% CI 4.8–10.9), and in STEP-HFpEF DM by 7.3 points (95% CI 4.1–10.4).

Pooled analysis using random-effects meta-analysis with Hartung-Knapp adjustment demonstrated a mean difference of + 7.4 points (95% CI 4.9–9.9), exceeding the 5-point threshold for clinically meaningful improvement. Between-study heterogeneity was minimal (I² = 0.0%, τ² = 0.00, Q = 0.09, *p* = 0.96), indicating highly consistent treatment effects across trials. The forest plot is shown in Supplementary Figure S2.

### Functional capacity (6-minute walk distance)

Three trials (SUMMIT, STEP-HFpEF, STEP-HFpEF DM) reported change in 6-minute walk distance at 52 weeks. In SUMMIT, tirzepatide improved 6MWD by 18.3 m (95% CI 9.9–26.7). In STEP-HFpEF, semaglutide improved 6MWD by 20.3 m (95% CI 8.6–32.1), and in STEP-HFpEF DM by 14.3 m (95% CI 3.7–24.9).

Pooled analysis using random-effects meta-analysis with Hartung-Knapp adjustment demonstrated a mean difference of + 17.6 m (95% CI 10.7–24.5). Between-study heterogeneity was minimal (I² = 0.0%, τ² = 0.00, Q = 0.61, *p* = 0.74), indicating consistent functional improvements across trials. The forest plot is shown in Supplementary Figure S3.

### Body weight

Three trials (SUMMIT, STEP-HFpEF, STEP-HFpEF DM) reported percentage change in body weight at 52 weeks. In SUMMIT, tirzepatide produced a weight reduction of -11.6% (95% CI -12.9 to -10.4). In STEP-HFpEF, semaglutide reduced weight by -10.7% (95% CI -11.9 to -9.4), and in STEP-HFpEF DM by -6.4% (95% CI -7.6 to -5.2).

Pooled analysis using random-effects meta-analysis with Hartung-Knapp adjustment demonstrated a mean difference of -9.6% (95% CI -11.3 to -8.0). Moderate heterogeneity was observed (I² = 45.8%, τ² = 3.62, Q = 3.69, *p* = 0.16), likely attributable to differences in baseline diabetes status and follow-up duration. The forest plot is shown in Supplementary Figure S4.

### All-cause mortality

Two trials (SUMMIT, SELECT HFpEF subgroup) reported all-cause mortality. In SUMMIT, mortality was numerically higher with tirzepatide (HR 1.25, 95% CI 0.63–2.45), while in SELECT HFpEF subgroup, mortality was numerically lower with semaglutide (HR 0.83, 95% CI 0.59–1.16).

Pooled analysis showed no statistically significant difference in all-cause mortality (HR 0.90, 95% CI 0.67–1.22); however, this finding should be interpreted as inconclusive. The opposing point estimates (HR 1.25 with tirzepatide in SUMMIT versus HR 0.83 with semaglutide in SELECT HFpEF), combined with 170 total deaths across 3,004 patients at median follow-up of 39.8 months in SELECT HFpEF and a median of 104 weeks in SUMMIT, indicate substantial underpowering for this endpoint. Absence of a statistically significant difference in this setting should not be interpreted as evidence of a null mortality effect. Between-study heterogeneity was low (I² = 18.5%, τ² = 0.03, Q = 1.23, *p* = 0.27). The forest plot is shown in Supplementary Figure S5.

### Safety

No consistent safety concerns were identified across trials. In STEP-HFpEF, serious adverse events occurred in 13.3% with semaglutide versus 26.7% with placebo (*p* < 0.001), driven primarily by fewer cardiac disorder events (2.7% vs. 11.3%). In STEP-HFpEF DM, serious adverse events occurred in 17.7% versus 28.8% (*p* = 0.002), again with fewer cardiac serious adverse events in the active arm (6.1% vs. 13.1%). In SUMMIT, serious adverse event rates were comparable between groups. Gastrointestinal adverse events were the most common cause of treatment discontinuation, occurring in 9.5–13.3% of active therapy recipients versus 2.0-5.3% in placebo groups across trials. No consistent signals for arrhythmia, ischemia, renal impairment, or hypotension were identified.

## Discussion

Across the four trials synthesized in this review—SUMMIT, STEP-HFpEF, STEP-HFpEF DM, and the HFpEF subgroup of SELECT—GLP-1 receptor agonists and dual GIP/GLP-1 agonists produced clinically meaningful improvements in patient-centered outcomes, functional capacity, body weight, and key HF endpoints. In the three HFpEF trials specifically designed around symptoms and function (SUMMIT, STEP-HFpEF, STEP-HFpEF DM), semaglutide and tirzepatide yielded 6-8-point improvements in KCCQ-CSS and 14-20-meter gains in 6MWD across individual trials, and a pooled improvement of 17.6 m (95% CI 10.7–24.5). While this falls below the conventional ≥ 30 m minimal clinically important difference established in general heart failure populations, it represents a statistically robust and consistent functional improvement. Some HFpEF-specific literature suggests lower thresholds may be appropriate given impaired baseline functional capacity in this phenotype, though we have not applied an alternative threshold without robust validation.

SELECT did not collect KCCQ or 6MWD, so its contribution is limited to HF outcomes rather than health-status measures. These findings reinforce the hypothesis that metabolic unloading, weight reduction, and improved cardiovascular efficiency directly contribute to symptomatic relief in HFpEF.

Across the two trials reporting adjudicated first heart failure hospitalization (SUMMIT and SELECT HFpEF subgroup), incretin therapy significantly reduced the risk of HF hospitalization. This pooled estimate is based on 82 adjudicated HHF events across 3,004 patients, and should be interpreted in that context. In the STEP-HFpEF trials, substantial reductions in adjudicated HF events (hospitalization or urgent HF visits) were also observed, although heterogeneity in endpoint definitions prevented pooling. Despite differences in trial design and definitions, all studies demonstrated a consistent direction of benefit across HF outcomes. Weight reduction was substantial across all HFpEF populations. Tirzepatide achieved approximately 12% greater weight loss than placebo in SUMMIT, while semaglutide achieved 10–11% reductions in STEP-HFpEF and 6–7% reductions in STEP-HFpEF DM. These magnitudes of weight loss align with the observed improvements in symptoms and function. Mortality remained inconclusive across available data. The divergent point estimates — HR 1.25 with tirzepatide in SUMMIT versus HR 0.83 with semaglutide in SELECT HFpEF — most plausibly reflect random variation given the small event counts rather than a genuine differential class effect. In SUMMIT, 19 deaths occurred in the tirzepatide arm versus 15 in placebo over a median of 104 weeks; in the SELECT HFpEF subgroup, 64 deaths occurred with semaglutide versus 72 with placebo over 39.8 months. Neither trial was powered for mortality as a standalone endpoint, and the pooled HR of 0.90 (95% CI 0.67–1.22) spans a range consistent with both meaningful benefit and modest harm. The follow-up duration in HFpEF-focused trials also may be insufficient to detect mortality differences that typically emerge over multi-year horizons with heart failure therapies. Ongoing outcomes trials in broader HF populations will be essential for resolving this question. Safety findings were reassuring. Gastrointestinal adverse events were common but typically mild, consistent with the known class profile of GLP-1-based therapies. Serious adverse events and treatment discontinuations were comparable or numerically lower with active therapy, and no concerning signals for arrhythmia, ischemia, renal impairment, or hypotension were detected.

Our meta-analysis revealed minimal heterogeneity across trials for most outcomes (I² = 0–19% for five of six outcomes), despite differences in trial design including varying LVEF thresholds (≥ 45% vs. ≥ 50%), follow-up durations (52 weeks to 39.8 months), and population characteristics (diabetes status, baseline functional impairment). Moderate heterogeneity was observed only for body weight change (I² = 45.8%), likely attributable to differences in diabetes status and trial duration, as STEP-HFpEF DM enrolled exclusively patients with type 2 diabetes and showed smaller weight reductions. We addressed heterogeneity using random-effects meta-analysis, with the Hartung-Knapp-Sidik-Jonkman adjustment applied to outcomes with three or more trials to provide appropriately conservative confidence intervals given the small number of studies.

The generalizability of these findings warrants careful consideration. Symptom and functional results (KCCQ-CSS, 6MWD) derive exclusively from trials requiring formal obesity criteria, structured HFpEF diagnostic criteria, and NYHA class II-IV symptoms. These findings therefore apply most directly to patients presenting with symptomatic obesity-related HFpEF in clinical practice and should not be assumed to generalize to lean or mildly overweight HFpEF phenotypes. The HHF signal derives from SUMMIT and SELECT HFpEF, which enrolled somewhat different populations: SUMMIT enriched for higher-risk patients by requiring recent HF decompensation or reduced renal function, while SELECT enrolled stable patients with established ASCVD. Clinicians applying these findings should consider whether their patients resemble these specific trial populations.

### Strengths

This systematic review has several strengths. We restricted inclusion to trials requiring obesity (BMI ≥ 30 kg/m²) as an entry criterion, focusing on a mechanistically distinct HFpEF phenotype rather than pooling heterogeneous cardiovascular outcome trials. We prespecified first adjudicated heart failure hospitalization as the primary outcome, providing concrete evidence for clinical decision-making. Unlike previous meta-analyses that included broader trials (SELECT, FLOW, EXSCEL) without specific obesity or HFpEF requirements, or analyzed composite/recurrent events, our approach targets the obesity-HFpEF population with a clinically actionable endpoint. We comprehensively assessed patient-centered outcomes including symptoms, functional capacity, and body weight alongside clinical events. The methodology followed PRISMA 2020 guidelines with duplicate screening, Cochrane risk of bias assessment, and GRADE evidence evaluation.

### Limitations

This systematic review has several limitations. First, only four randomized trials met inclusion criteria, limiting the precision of pooled estimates, particularly for mortality. Furthermore, the primary outcome meta-analysis rests on 82 adjudicated HHF events across 3,004 patients, limiting the precision of the pooled estimate and underscoring the need for replication in future dedicated HFpEF outcomes trials. Second, heterogeneity in outcome definitions prevented pooling of all HF events; STEP-HFpEF trials reported HF events using a broader endpoint definition that incorporated urgent IV visits in addition to hospitalizations, preventing direct pooling with SUMMIT and SELECT HFpEF. Third, while the SELECT HFpEF analysis was prespecified, SELECT was designed and powered for MACE reduction rather than HF hospitalization in an HFpEF subgroup, and the HHF HR from this source crossed unity. Additionally, approximately 24% of SELECT HFpEF subgroup participants had BMI 27–29.9 kg/m², qualifying under our prespecified ≥ 27 + comorbidity criterion rather than the primary ≥ 30 threshold. This introduces modest population heterogeneity, though the majority of participants in this subgroup met BMI ≥ 30. Fourth, the SELECT HFpEF subgroup was not prespecified for all outcomes, and weight change data were unavailable for this cohort. Fifth, follow-up duration was relatively short (52 weeks in HFpEF-focused trials), potentially insufficient to detect long-term mortality differences or durability of treatment effects. Sixth, our findings are specific to obesity-related HFpEF and may not generalize to lean or mildly overweight HFpEF phenotypes. Finally, only semaglutide and tirzepatide have been evaluated in HFpEF trials; effects of other GLP-1 agonists remain unknown.

Taken together, this systematic review highlights the consistent, clinically meaningful benefits of GLP-1 receptor agonists and dual GIP/GLP-1 agonists in obesity-related HFpEF. Improvements in symptoms, exercise capacity, HF events, and substantial weight loss collectively support a metabolic-directed therapeutic strategy. Future research should evaluate long-term cardiovascular outcomes, interactions with SGLT2 inhibitors, and mechanistic pathways linking metabolic improvement to HFpEF progression.

## Conclusion

In adults with heart failure with preserved ejection fraction and obesity, GLP-1 receptor agonists and dual GIP/GLP-1 agonists consistently improve symptoms, physical function, and body weight. Across HFpEF-focused randomized trials, semaglutide and tirzepatide produced clinically meaningful gains in KCCQ-CSS, exercise capacity, and substantial weight reduction. Trials reporting heart failure events demonstrated favorable effects on HF hospitalization and composite HF outcomes, while mortality remained inconclusive.

## Supplementary Information

Supplementary Material 1: Figure S1. Forest plot for first adjudicated heart failure hospitalization. Random-effects meta-analysis of two randomized controlled trials (SUMMIT and SELECT HFpEF subgroup; *n*=3,004) comparing incretin-based therapies versus placebo on first adjudicated heart failure hospitalization in adults with obesity-related heart failure with preserved ejection fraction. Each trial is represented by a square (size proportional to weight in meta-analysis) with horizontal lines indicating 95% confidence intervals. The pooled hazard ratio (diamond) was 0.52 (95% CI 0.33-0.82; *p*=0.005), demonstrating a significant 48% relative risk reduction. Between-study heterogeneity was minimal (I²=0.0%, τ²=0.00, Q=0.48, *p*=0.49). Statistical analysis used inverse variance weighting with the DerSimonian-Laird random-effects model. CI, confidence interval; HR, hazard ratio; HFpEF, heart failure with preserved ejection fraction; SELECT, Semaglutide Effects on Cardiovascular Outcomes in People with Overweight or Obesity; SUMMIT, Study of Tirzepatide in Participants with Heart Failure with Preserved Ejection Fraction and Obesity. Figure S2. Forest plot for Kansas City Cardiomyopathy Questionnaire Clinical Summary Score. Random-effects meta-analysis of three randomized controlled trials (SUMMIT, STEP-HFpEF, STEP-HFpEF DM; *n*=1,876) evaluating the effect of incretin-based therapies versus placebo on change from baseline in Kansas City Cardiomyopathy Questionnaire Clinical Summary Score (KCCQ-CSS) at 52 weeks in patients with obesity-related HFpEF. Each trial is represented by a square with horizontal lines indicating 95% confidence intervals. The pooled mean difference (diamond) was +7.4 points (95% CI 4.9-9.9; *p*<0.001), exceeding the 5-point threshold for clinically meaningful improvement. Heterogeneity was minimal (I²=0.0%, τ²=0.00, Q=0.09, *p*=0.96). Analysis used the Hartung-Knapp adjustment for random-effects meta-analysis. KCCQ-CSS scores range from 0-100, with higher scores indicating better health status. CI, confidence interval; HFpEF, heart failure with preserved ejection fraction; STEP-HFpEF, Effect of Semaglutide on Function and Symptoms in Subjects with Obesity-related HFpEF; STEP-HFpEF DM, Semaglutide Treatment Effect in People with Obesity and HFpEF and Diabetes Mellitus. Figure S3. Forest plot for six-minute walk distance. Random-effects meta-analysis of three randomized controlled trials (SUMMIT, STEP-HFpEF, STEP-HFpEF DM; *n*=1,876) evaluating change from baseline in six-minute walk distance at 52 weeks with incretin-based therapies versus placebo in patients with obesity-related HFpEF. The pooled mean difference (diamond) was +17.6 meters (95% CI 10.7-24.5; *p*<0.001). Increases of ≥30 meters are considered clinically meaningful by conventional general heart failure criteria; the pooled estimate of 17.6 m falls below this threshold but represents a statistically significant and consistent functional improvement. Heterogeneity was minimal (I²=0.0%, τ²=0.00, Q=0.61, *p*=0.74). The six-minute walk test is a validated measure of submaximal functional exercise capacity in heart failure patients. Analysis used the Hartung-Knapp adjustment for random-effects meta-analysis with inverse variance weighting. CI, confidence interval; HFpEF, heart failure with preserved ejection fraction; STEP-HFpEF, Effect of Semaglutide on Function and Symptoms in Subjects with Obesity-related HFpEF; STEP-HFpEF DM, Semaglutide Treatment Effect in People with Obesity and HFpEF and Diabetes Mellitus; SUMMIT, Study of Tirzepatide in Participants with HFpEF and Obesity. Figure S4. Forest plot for percentage body weight change. Random-effects meta-analysis of three randomized controlled trials (SUMMIT, STEP-HFpEF, STEP-HFpEF DM; n=1,876) evaluating percentage change in body weight from baseline to 52 weeks with incretin-based therapies versus placebo in patients with obesity-related HFpEF. The pooled mean difference (diamond) was -9.6% (95% CI -11.3 to -8.0; *p*<0.001), indicating substantial weight loss. Moderate heterogeneity was observed (I²=45.8%, τ²=3.62, Q=3.69, *p*=0.16), likely attributable to differences in baseline diabetes status (STEP-HFpEF enrolled patients without diabetes, while STEP-HFpEF DM required type 2 diabetes). Analysis used the Hartung-Knapp adjustment for random-effects meta-analysis. Weight loss of ≥5% is considered clinically meaningful for obesity-related conditions. CI, confidence interval; HFpEF, heart failure with preserved ejection fraction; STEP-HFpEF, Effect of Semaglutide on Function and Symptoms in Subjects with Obesity-related HFpEF; STEP-HFpEF DM, Semaglutide Treatment Effect in People with Obesity and HFpEF and Diabetes Mellitus; SUMMIT, Study of Tirzepatide in Participants with HFpEF and Obesity. Figure S5. Forest plot for all-cause mortality. Random-effects meta-analysis of two randomized controlled trials (SUMMIT and SELECT HFpEF subgroup; *n*=3,004) evaluating all-cause mortality with incretin-based therapies versus placebo in patients with obesity-related HFpEF. The pooled hazard ratio (diamond) was 0.90 (95% CI 0.67-1.22; *p*=0.50), indicating no significant difference in mortality risk. Heterogeneity was low (I²=18.5%, τ²=0.03, Q=1.23, *p*=0.27). The relatively short follow-up duration in HF-focused trials (median 104 weeks in SUMMIT) may have been insufficient to detect mortality differences, as mortality benefits of HF therapies often emerge after prolonged follow-up. Statistical analysis used inverse variance weighting with the DerSimonian-Laird random-effects model. CI, confidence interval; HR, hazard ratio; HFpEF, heart failure with preserved ejection fraction; SELECT, Semaglutide Effects on Cardiovascular Outcomes in People with Overweight or Obesity; SUMMIT, Study of Tirzepatide in Participants with Heart Failure with Preserved Ejection Fraction and Obesity. Figure S6. Forest plot for composite cardiovascular death or heart failure events. Random-effects meta-analysis of two randomized controlled trials (SUMMIT and SELECT HFpEF subgroup; *n*=3,004) evaluating the composite outcome of cardiovascular death or worsening heart failure events with incretin-based therapies versus placebo in adults with obesity-related HFpEF. The pooled hazard ratio (diamond) was 0.69 (95% CI 0.53-0.91; *p*=0.008), demonstrating a significant 31% relative risk reduction. Heterogeneity was minimal (I²=0.0%, τ²=0.00, Q=0.47, *p*=0.49). This composite endpoint represents a clinically meaningful combination of hard cardiovascular outcomes. Heart failure events were defined as adjudicated hospitalizations or urgent visits requiring intravenous therapy. Statistical analysis used inverse variance weighting with the DerSimonian-Laird random-effects model. CI, confidence interval; HR, hazard ratio; HFpEF, heart failure with preserved ejection fraction; SELECT, Semaglutide Effects on Cardiovascular Outcomes in People with Overweight or Obesity; SUMMIT, Study of Tirzepatide in Participants with Heart Failure with Preserved Ejection Fraction and Obesity. CI = confidence interval; HR = hazard ratio; KCCQ-CSS = Kansas City Cardiomyopathy Questionnaire Clinical Summary Score; MD = mean difference.

## Data Availability

All data generated or analyzed during this study are included in this published article and its supplementary information files.
