# Supplementary material for: GLP-1 and dual GIP/GLP-1 agonists in obese patients with HFpEF: a systematic review and meta-analysis of RCTs
Source: BMC Cardiovasc Disord. 2026 Mar 30;26:406. doi: 10.1186/s12872-026-05808-7 (PMC13154893; doi:10.1186/s12872-026-05808-7)

# Supplementary Appendix 1. PRISMA 2020 Flow Diagram

**Identification of studies via databases and registers**

Records removed *before screening*:

Duplicate records removed (n = 127)

Records marked as ineligible by automation tools (n = 0)

Records removed for other reasons (n = 0)

Records identified from*:

Databases (n = 387)

Registers (n = 20)

Hand-searching prominent Scopus-indexed cardiology journals (n = 12)

**Identification**

Records screened

(n = 292)

Records excluded**

(n = 287)

Reports sought for retrieval

(n = 5)

Reports not retrieved

(n = 0)

**Screening**

Reports assessed for eligibility

(n = 5)

Reports excluded (n = 1):

• Wrong population; prespecified subgroup from same study included (n = 1)

Studies included in review

(n = 4)

**Included**

*Consider, if feasible to do so, reporting the number of records identified from each database or register searched (rather than the total number across all databases/registers).

**If automation tools were used, indicate how many records were excluded by a human and how many were excluded by automation tools.

Source: Page MJ, et al. BMJ 2021;372:n71. doi: 10.1136/bmj.n71.

This work is licensed under CC BY 4.0. To view a copy of this license, visit <https://creativecommons.org/licenses/by/4.0/>

# Appendix 2. Full Search Strategy

Date of Search: November 2025

Objective of Search Strategy:

To identify all randomized controlled trials evaluating GLP-1 receptor agonists and dual GLP-1/GIP receptor agonists in patients with HFpEF, The search was limited to two core concepts: (1) GLP-1 receptor agonists and dual GLP-1/GIP receptor agonists, and (2) HFpEF. No filters for outcomes, obesity, or study design were applied at the search stage to maximize sensitivity; these were applied during screening.

Search Strings by Source:

1.PubMed / MEDLINE:

("Glucagon-Like Peptide 1 Receptor Agonists"[Mesh] OR semaglutide[tiab] OR tirzepatide[tiab] OR GLP-1[tiab] OR "GLP-1 receptor agonist*"[tiab])

AND

("Heart Failure, Diastolic"[Mesh] OR HFpEF[tiab] OR "heart failure with preserved ejection fraction"[tiab] OR "diastolic heart failure"[tiab])

2. Cochrane CENTRAL:

("GLP-1 receptor agonist*" OR "glucagon-like peptide 1" OR semaglutide

OR tirzepatide):ti,ab,kw

AND

(HFpEF OR "heart failure with preserved ejection fraction"):ti,ab,kw

3. Journal Websites (e.g., Circulation, European Heart Journal, Nature Reviews

Cardiology, Journal of the American College of Cardiology, JAMA Cardiology):

(GLP-1 OR semaglutide OR tirzepatide OR liraglutide) AND (HFpEF OR "heart failure with preserved

ejection fraction")

4.Clinical Trials Registries (ClinicalTrials.gov / ICTRP):

(GLP-1 OR semaglutide OR tirzepatide) AND (HFpEF OR "heart failure with preserved ejection fraction")

5. Epistemonikos:

("GLP-1" OR semaglutide OR tirzepatide) AND (HFpEF OR "heart failure with preserved ejection fraction")

# Appendix 3. Risk of Bias Assessment (RoB 2)

Risk of bias was assessed using the Cochrane Risk of Bias 2 (RoB 2) tool across five domains: (1) randomization process, (2) deviations from intended interventions, (3) missing outcome data, (4) measurement of the outcome, and (5) selection of the reported result.

| Trial | D1 Randomization | D2 Deviations | D3 Missing data | D4 Measurement | D5 Reported result | Overall RoB |
| --- | --- | --- | --- | --- | --- | --- |
| SUMMIT | Some concerns | Low risk | Low risk | Low risk | Some concerns | Some concerns |
| STEP-HFpEF | Some concerns | Low risk | Low risk | Low risk | Low risk | Some concerns |
| STEP-HFpEF DM | Some concerns | Low risk | Low risk | Low risk | Low risk | Some concerns |
| SELECT HFpEF subgroup | Low risk | Low risk | Low risk | Low risk | Low risk | Low risk |

Note: "Some concerns" in the randomization domain for SUMMIT and STEP trials reflects limited reporting of sequence generation and allocation concealment in the primary publications, despite generally balanced baseline characteristics and double-blind design. SELECT was judged low risk in all domains.

# Appendix 3B. Risk of Bias Justification (RoB 2)

## SUMMIT

**D1:** Randomization sequence generation and allocation concealment not fully described in main NEJM article. Supplementary materials mention centralized randomization but without methodological detail → Some concerns.

**D2:** Double-blind, identical injections → Low risk.

**D3:** Low missing data; follow-up >95% → Low risk.

**D4:** Blinded adjudication of HF outcomes → Low risk.

**D5:** Primary/secondary endpoints prespecified but SAP partially summarized only → Some concerns.

## STEP-HFpEF

**D1:** Randomization described as centralized/stratified but without details → Some concerns.

**D2:** Double-blind, consistent exposure → Low risk.

**D3:** Minimal missing data for primary outcomes → Low risk.

**D4:** Standardized measurement (KCCQ, 6MWD) → Low risk.

**D5:** Prespecified endpoints, hierarchy respected → Low risk.

## STEP-HFpEF DM

**D1:** Same limitations as STEP-HFpEF regarding incomplete reporting → Some concerns.

**D2:** Double-blind, consistent intervention → Low risk.

**D3:** Missing data minimal → Low risk.

**D4:** Validated outcome measures, blinded adjudication for HF → Low risk.

**D5:** No selective reporting detected → Low risk.

## SELECT HFpEF subgroup

**D1:** Large RCT with robust central randomization described fully → Low risk.

**D2:** Double-blind design, strict protocol adherence → Low risk.

**D3:** Excellent completeness of follow-up → Low risk.

**D4:** Independent event adjudication → Low risk.

**D5:** Prespecified HF endpoints in subgroup → Low risk.

# Appendix 4. Extracted Outcome Data from Included Trials

# **This appendix summarizes the key extracted outcome data used in the quantitative synthesis. Effect estimates are presented as reported in the trials**

| Trial | | Outcome | Measure | Effect estimate (95% CI) | Notes |
| --- | --- | --- | --- | --- | --- |
| SUMMIT | | First HF hospitalization | HR | 0.44 (0.22–0.87) | Tirzepatide vs placebo; HFpEF with obesity |
| SUMMIT | | KCCQ-CSS change | MD (points) | +6.9 (3.3–10.6) | Change from baseline at ~52 weeks |
| SUMMIT | | 6MWD change | MD (m) | +18.3 (9.9–26.7) | Change from baseline at ~52 weeks |
| SUMMIT | | Weight (% change) | MD (%) | −11.6 (−12.9 to −10.4) | Placebo-corrected % change |
| SUMMIT | | All-cause mortality | HR | 1.25 (0.63–2.45) | Few events; wide CI |
| SUMMIT | | Composite CV death or worsening HF | HR | 0.62 (0.41–0.95) | Primary composite endpoint |
| STEP-HFpEF | | KCCQ-CSS change | MD (points) | +7.8 (4.8–10.9) | Semaglutide 2.4 mg vs placebo; 52 weeks |
| STEP-HFpEF | 6MWD change | | MD (m) | +20.3 (8.6–32.1) | Change from baseline at 52 weeks |
| STEP-HFpEF | Weight (% change) | | MD (%) | −10.7 (−11.9 to −9.4) | Placebo-corrected % change |
| STEP-HFpEF | HF events (hospitalization/urgent visit) | | HR (composite) | Not used for HHF meta-analysis | Reported as composite and hierarchical win-ratio |
| STEP-HFpEF DM | KCCQ-CSS change | | MD (points) | +7.3 (4.1–10.4) | Semaglutide 2.4 mg vs placebo; 52 weeks |
| STEP-HFpEF DM | 6MWD change | | MD (m) | +14.3 (3.7–24.9) | Change from baseline at 52 weeks |
| STEP-HFpEF DM | Weight (% change) | | MD (%) | −6.4 (−7.6 to −5.2) | Placebo-corrected % change |
| STEP-HFpEF DM | HF events (hospitalization/urgent visit) | | HR (composite) | Not used for HHF meta-analysis | Reported as composite and hierarchical win-ratio |
| SELECT HFpEF subgroup | First HF hospitalization | | HR | 0.59 (0.31–1.06) | Semaglutide 2.4 mg vs placebo; HFpEF subgroup |
| SELECT HFpEF subgroup | HF composite (CV death or HF event) | | HR | 0.75 (0.52–1.07) | HFpEF-specific composite outcome |
| SELECT HFpEF subgroup | All-cause mortality | | HR | 0.83 (0.59–1.16) | HFpEF subgroup |
| SELECT HFpEF subgroup | Cardiovascular death | | HR | 0.87 (0.56–1.34) | HFpEF subgroup |

# Appendix 5A. GRADE Summary of Findings Table

| Outcome | Effect (95% CI) | No. of Trials | Certainty (GRADE) | Comments |
| --- | --- | --- | --- | --- |
| First HF hospitalization | HR 0.52 (0.33–0.83) | 2 | Moderate | Downgraded for imprecision; consistent direction of effect. |
| Composite CV death/HF events | HR 0.69 (0.53–0.91) | 2 | Moderate | Driven mainly by HF events; robust effect. |
| KCCQ-CSS change | MD +7.4 points | 3 | High | Consistent, precise, clinically significant. |
| 6MWD improvement | MD +17.6 m | 3 | High | Consistent across diabetic and non-diabetic HFpEF. |
| Weight change (%) | MD −9.5% | 3 | High | Large, consistent effect. |
| All-cause mortality | HR 0.90 (0.67–1.22) | 2 | Moderate | Downgraded for imprecision; no harm. |
| Cardiovascular mortality | HR 0.93 (0.64–1.34) | 2 | Low–Moderate | Imprecision + low event count. |

# Appendix 5B. Detailed GRADE Evidence Profile

## First HF hospitalization

**risk:** No serious concerns

**inconsistency:** No serious inconsistency (I²≈0%)

**indirectness:** Direct evidence in obese HFpEF

**imprecision:** Some imprecision (event counts modest)

**publication:** No concerns

**certainty:** Moderate

## Composite CV death/HF

**risk:** Low risk of bias trials

**inconsistency:** Low heterogeneity

**indirectness:** Direct evidence

**imprecision:** Moderate

**publication:** No concerns

**certainty:** Moderate

## KCCQ-CSS

**risk:** Low

**inconsistency:** Consistent across trials

**indirectness:** Direct

**imprecision:** None (tight CI)

**publication:** No concerns

**certainty:** High

## 6MWD

**risk:** Low

**inconsistency:** Minimal

**indirectness:** Direct

**imprecision:** Low

**publication:** No concerns

**certainty:** High

## Weight change

**risk:** Low

**inconsistency:** Some (diabetes subgroup attenuated effect)

**indirectness:** Direct

**imprecision:** Low

**publication:** No concerns

**certainty:** High

## All-cause mortality

**risk:** Low

**inconsistency:** Low

**indirectness:** Direct

**imprecision:** Serious (wide CI)

**publication:** No concerns

**certainty:** Moderate

## CV mortality

**risk:** Low

**inconsistency:** Low

**indirectness:** Direct

**imprecision:** Serious

**publication:** No concerns

**certainty:** Low–Moderate

## Appendix 6. Forest Plots

## Figure S1. HF Hospitalization


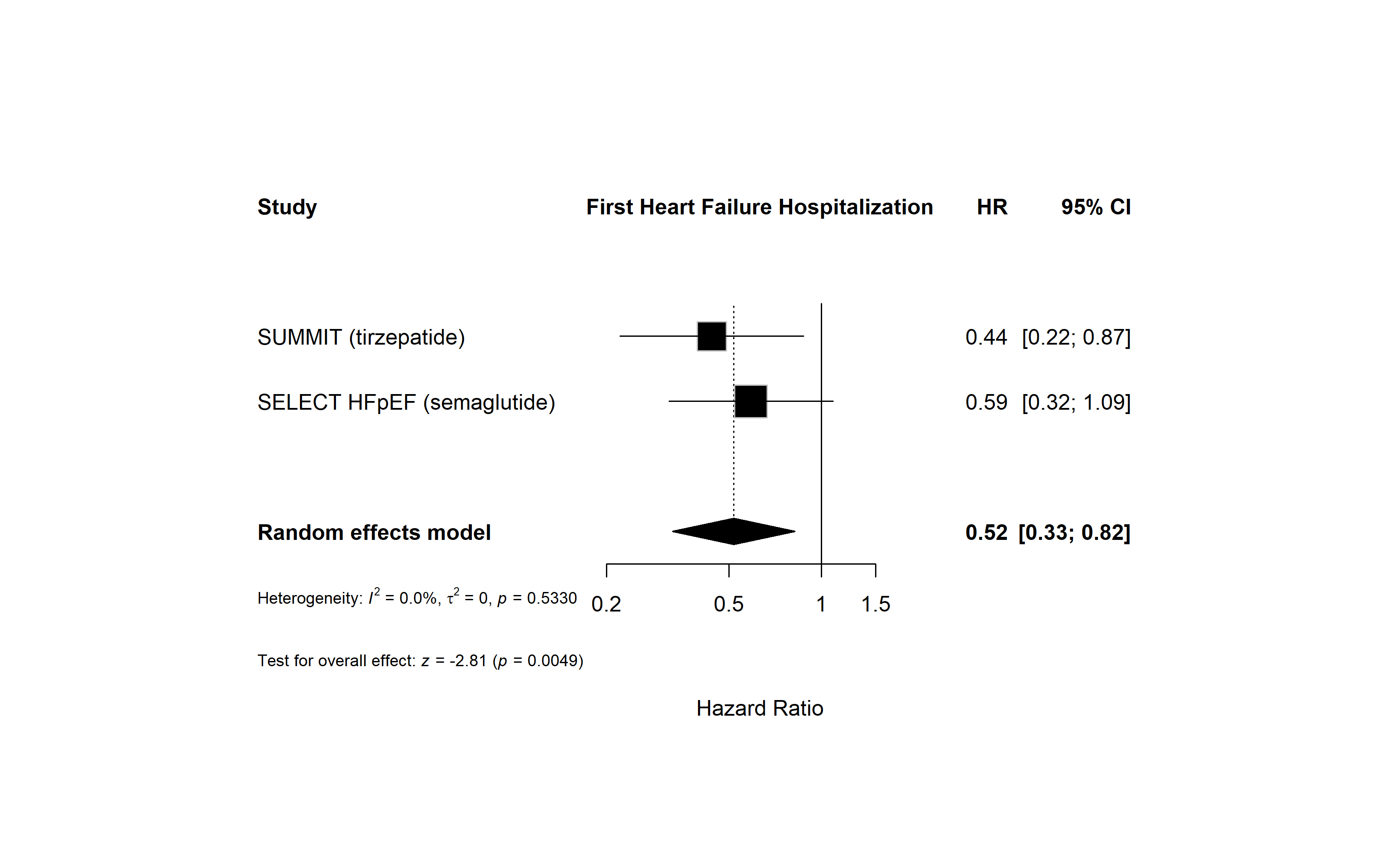


Figure.S2 .KCCQ-CSS


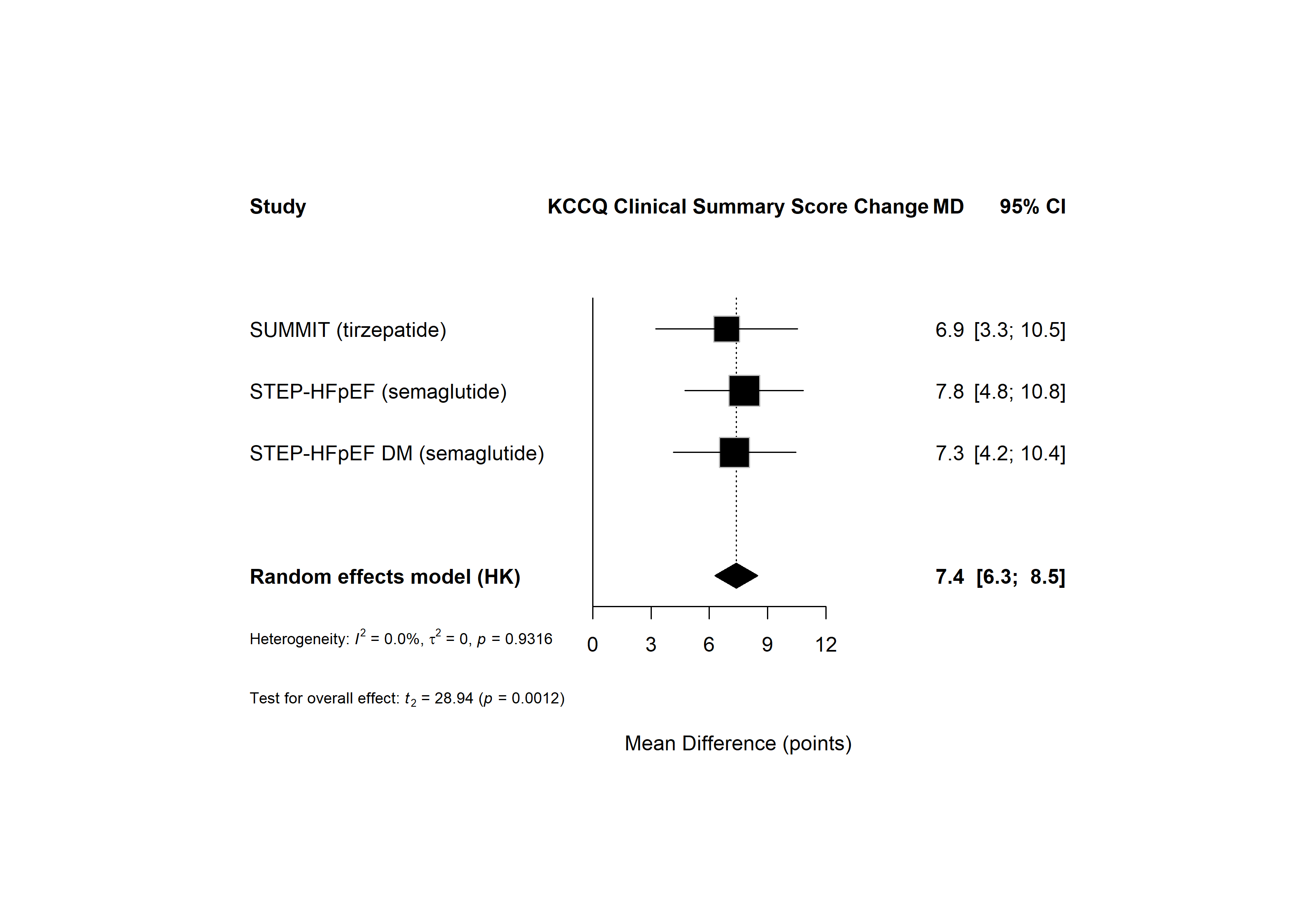


Figure S3. 6MWD


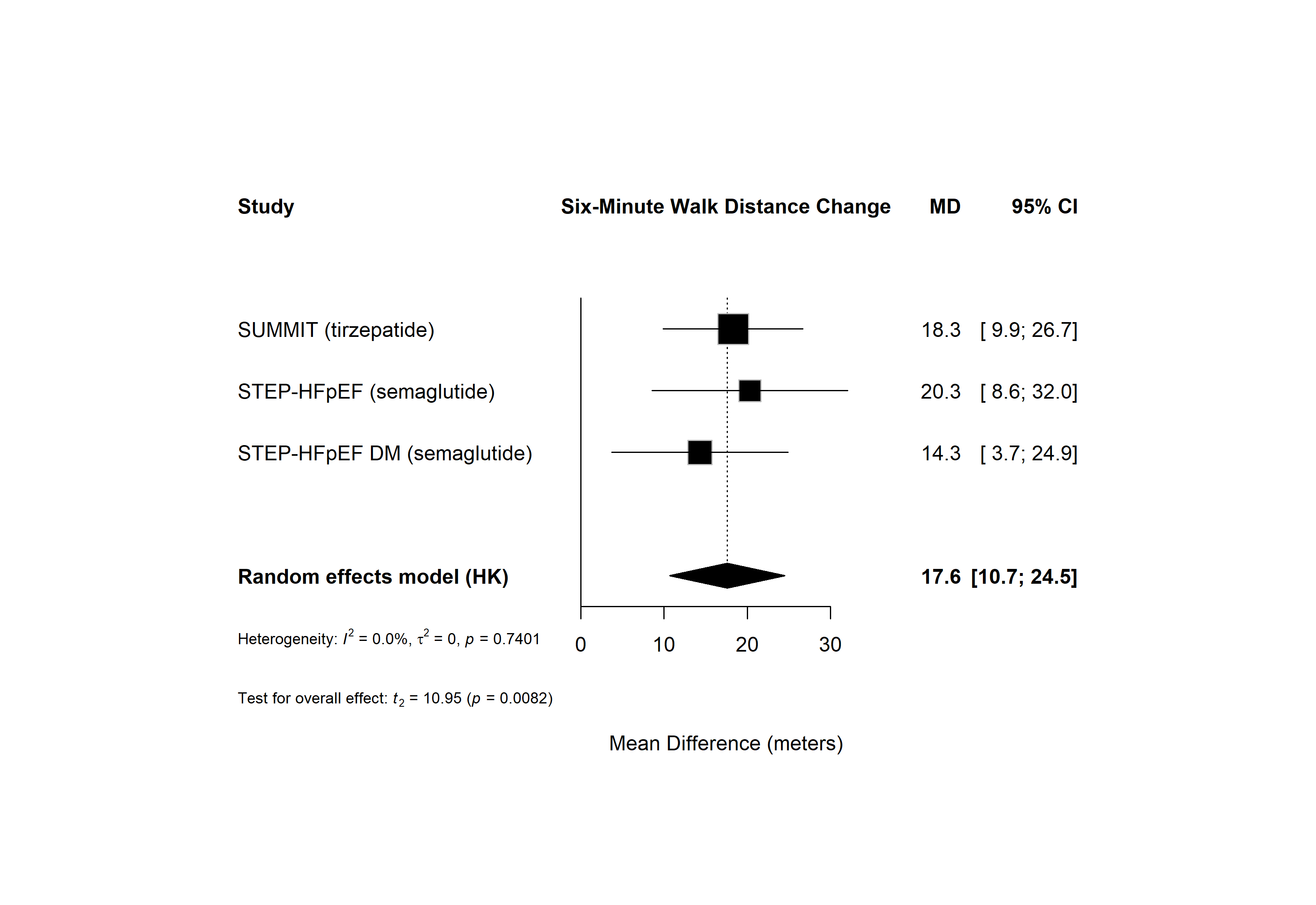


Figure S4. Weight


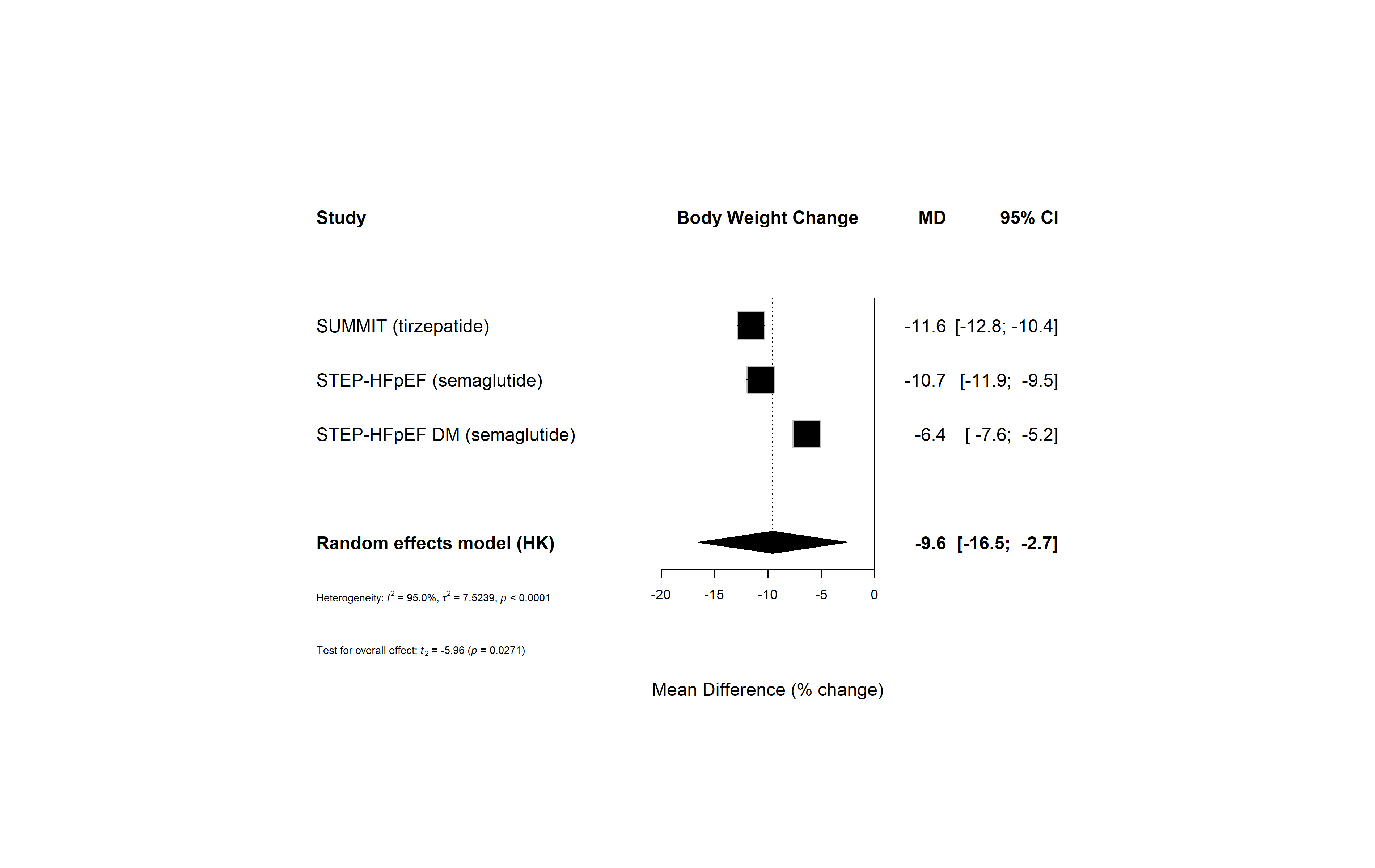


Figure S5. All-Cause Mortality


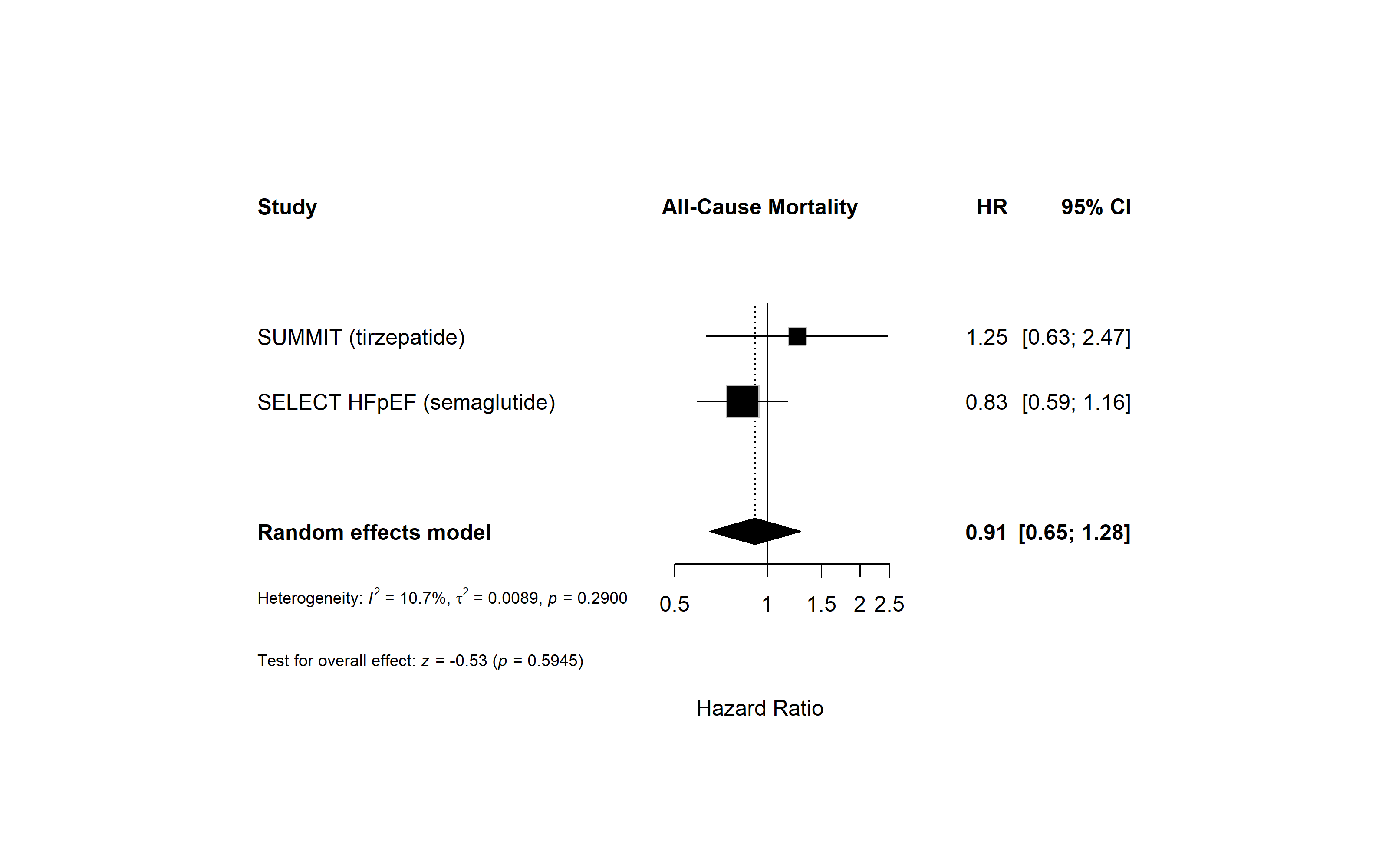


Figure S6. Composite CV Death/HF Events


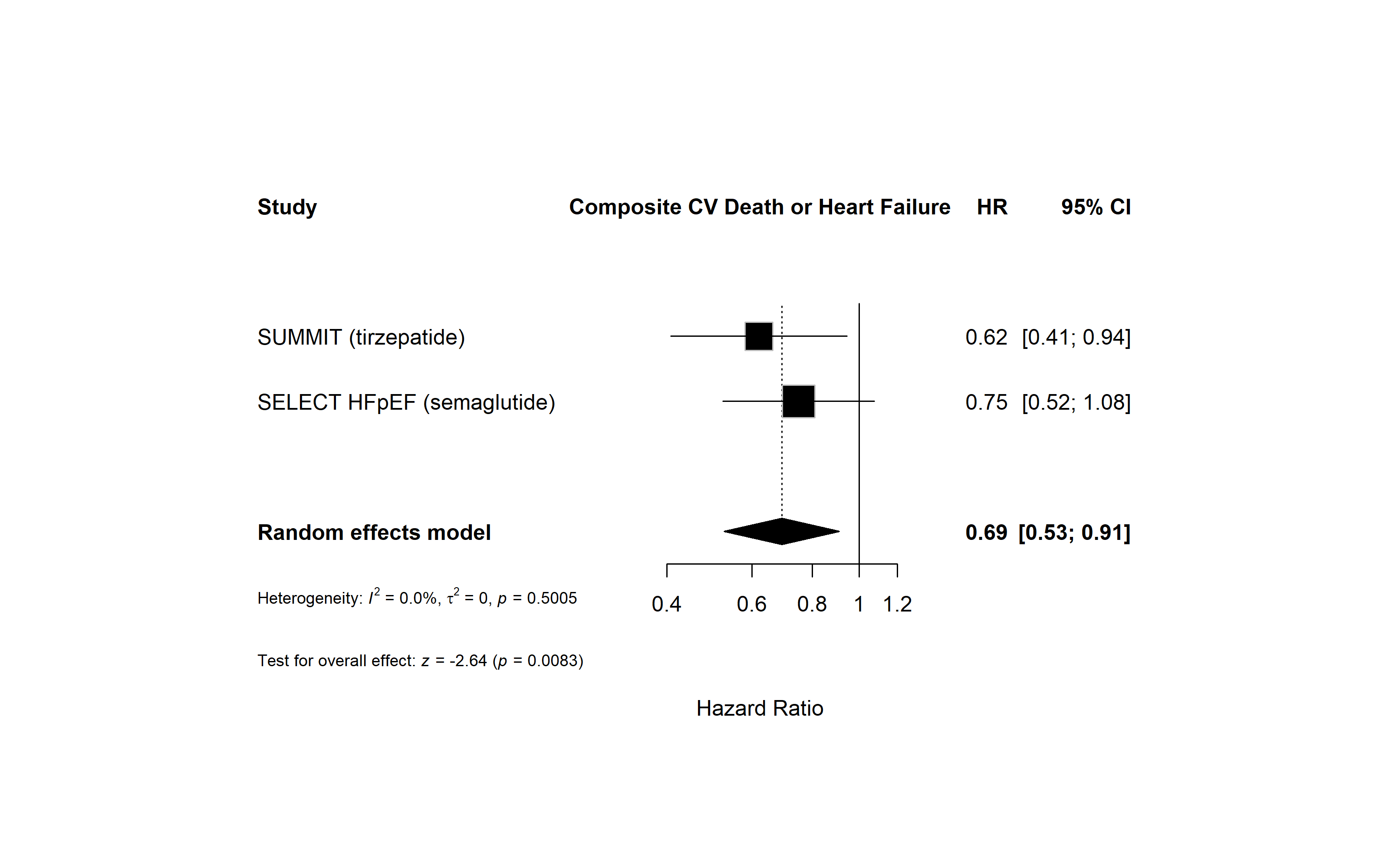

Supplement: Supplementary file 1 — Supplementary Material 1: Figure S1. Forest plot for first adjudicated heart failure hospitalization. Random-effects meta-analysis of two randomized controlled trials (SUMMIT and SELECT HFpEF subgroup; n=3,004) comparing incretin-based therapies versus placebo on first adjudicated heart failure hospitalization in adults with obesity-related heart failure with preserved ejection fraction. Each trial is represented by a square (size proportional to weight in meta-analysis) with horizontal lines indicating 95% confidence intervals. The pooled hazard ratio (diamond) was 0.52 (95% CI 0.33-0.82; p=0.005), demonstrating a significant 48% relative risk reduction. Between-study heterogeneity was minimal (I²=0.0%, τ²=0.00, Q=0.48, p=0.49). Statistical analysis used inverse variance weighting with the DerSimonian-Laird random-effects model. CI, confidence interval; HR, hazard ratio; HFpEF, heart failure with preserved ejection fraction; SELECT, Semaglutide Effects on Cardiovascular Outcomes in People with Overweight or Obesity; SUMMIT, Study of Tirzepatide in Participants with Heart Failure with Preserved Ejection Fraction and Obesity. Figure S2. Forest plot for Kansas City Cardiomyopathy Questionnaire Clinical Summary Score. Random-effects meta-analysis of three randomized controlled trials (SUMMIT, STEP-HFpEF, STEP-HFpEF DM; n=1,876) evaluating the effect of incretin-based therapies versus placebo on change from baseline in Kansas City Cardiomyopathy Questionnaire Clinical Summary Score (KCCQ-CSS) at 52 weeks in patients with obesity-related HFpEF. Each trial is represented by a square with horizontal lines indicating 95% confidence intervals. The pooled mean difference (diamond) was +7.4 points (95% CI 4.9-9.9; p<0.001), exceeding the 5-point threshold for clinically meaningful improvement. Heterogeneity was minimal (I²=0.0%, τ²=0.00, Q=0.09, p=0.96). Analysis used the Hartung-Knapp adjustment for random-effects meta-analysis. KCCQ-CSS scores range from 0-100, with higher s [file 12872_2026_5808_MOESM1_ESM.docx]
